# Chemical Composition and Evaluation of Antifungal and Insecticidal Activities of Essential Oils Extracted from *Jambosa caryophyllus* (Thunb.) Nied: Clove Buds

**DOI:** 10.1155/2022/4675016

**Published:** 2022-10-19

**Authors:** Otmane Zouirech, Reem Alajmi, Hiba El jeddab, Aimad Allali, Mohammed Bourhia, Abdelfattah El Moussaoui, Azeddin El Barnossi, Ashraf M. Ahmed, John P. Giesy, Mourad A. M. Aboul-Soud, Badiaa Lyoussi, El houssine Derwich

**Affiliations:** ^1^Laboratory of Natural Substances, Pharmacology Environment, Modeling, Health and Quality of Life, Faculty of Sciences Dhar El Mahraz, University Sidi Mohamed Ben Abdellah, Fez, Morocco; ^2^Department of Zoology, College of Science King Saud University, Riyadh 11451, Saudi Arabia; ^3^Laboratory of Animal and Plant Production, and Agro-Industry, Faculty of Sciences, Ibn Tofail University, BP 133, Kenitra 14000, Morocco; ^4^Higher Institute of Nursing Professions and Technical Health, Laayoune 70000, Morocco; ^5^Laboratory of Biotechnology, Environment, Agrifood and Health, Faculty of Science, University of Sidi Mohamed Ben Abdellah, Fez 30050, Morocco; ^6^Toxicology Centre, University of Saskatchewan, SK S7N 5B3, Saskatoon, Canada; ^7^Department of Veterinary Biomedical Sciences, University of Saskatchewan, SK S7N 5B4, Saskatoon, Canada; ^8^Department of Integrative Biology, Michigan State University, East Lansing 48824, MI, USA; ^9^Department of Environmental Sciences, Baylor University, Waco 76706, TX, USA; ^10^Department of Clinical Laboratory Sciences, College of Applied Medical Sciences, King Saud University, P.O. Box 10219, Riyadh 11433, Saudi Arabia; ^11^Unity of GC/MS and GC-FID, City of Innovation, Sidi Mohamed Ben Abdallah University, Fez, Morocco

## Abstract

*Jambosa caryophyllus* has been used in traditional phytotherapy as a treatment against infections. In the present work, essential oils extracted from clove buds (*Jambosa caryophyllus ***)** (EO-JC) were investigated for their composition, antifungal, and insecticidal properties. Extraction of EO-JC was performed by use of hydrodistillation using a Clevenger-type apparatus, and the EOs were analyzed by gas chromatography coupled with mass spectrometry (GC-MS). Antifungal activity of EO-JC was evaluated by the use of solid-state diffusion (disc method) and microdilution to determine the minimum inhibitory concentration (MIC), against three strains of fungus, *Aspergillus niger*, *Aspergillus flavus,* and *Fusarium oxysporum*. Insecticidal activity of EO-JC against the cowpea weevil, *Callosobruchus maculatus*, was determined to assess utility of EO-JC to control this pest. Several exposures including inhalation and contact were used to determine lethality, as well as the repulsion test was conducted at concentrations of 4, 8, 16, and 32 *μ*L EO-JC. Characterization of EO-JC by GC/MS revealed 34 compounds accounting for 99.98% of the mass of the extract. The predominant compound was eugenol (26.80%) followed by *β*-caryophyllene (16.03%) and eugenyl acetate (5.83%). The antifungal activity of EO-JC on solid media exhibited inhibitions in the range of 49% to 87%, and MIC was between 3.125 and 7.80 *μ*g EO-JC/mL. Insecticidal activity, as determined by the use of the inhalation test, and expressed as the LD_50_ and LD_95_ after 96 hours of exposure was 2.32 and 21.92 *μ*L/L air, respectively. In the contact test, a 96-hour exposure resulted in LD_50_ and LD_95_ of 5.51 and 11.05 *μ*L/L of air, respectively. EO-JC exhibited insecticidal activity against fungi and pest chickpea weevil.

## 1. Introduction

Chickpea (Cicer arietinum L.) is a very important crop in Morocco's production systems, coming in second place after fava beans with an area of 59,000 ha in 2014–2015 (MAPM's annual report 2016) [1]. It is an important source of carbohydrates and proteins, contains a significant amount of all essential amino acids, and is an important source of important vitamins such as riboflavin, niacin, thiamine, folate, and the precursor of vitamin A, *β*-carotene [2]. However, it is attacked by several insects of which the most dreaded is Callosobruchus maculatus, a beetle, in the leaf cutter family, Chrysomelidae which is among the most important species of insect pests of food legumes and is responsible for significant losses by reducing the quality and/or quantity of stored chickpea seed yield products during storage [3, 4].

The control of insects and other pests in warehouses has been adequately controlled by chemical control methods, including fumigation of stored products with carbon disulfide, phosphine, or spraying with malathion, carbaryl, pirimiphos-methyl, or permethrin. These chemicals have been reported to be effective against *C. maculatus* and other insect pests. However, uses of synthetic insecticides raise problems related to their cost as well as concerns for health of humans and effects on the environmental, as well as development of resistance to insecticides [5, 6]. These dangers have pushed the WHO to ban the use of certain chemical insecticides from more than one chemical family (organochlorine compounds, carbamates and thiocarbamates, organophosphates, and synthetic pyrethroids) [7, 8]. Hence, in order to avoid synthetic insecticides, the importance of obtaining effective natural alternatives that are cost effective, easily applicable, of minimal toxic potency to humans and wildlife, nonpersistent, biodegradable, and environmentally benign [9].

Due to adverse effects on both quality and quantity, exposure of plants and preserve seeds to fungal pathogens can lead to considerable economic losses. Various fungal contaminants can cause considerable losses and attack chickpea seeds and other foodstuffs. In particular, *Aspergillus niger* is one of the fungi most frequently isolated from peanuts, pecans, pistachios, hazelnuts, walnuts, kola nuts, coconuts, and copra [10]. *Aspergillus flavus* is a saprotrophic and pathogenic fungus of cosmopolitan distribution. It is best known for its colonization of cereals, legumes, and the fungus [11], *Fusarium oxysporum* f. sp. *albedinis* is the agent of vascular wilt of date palm (*Phoenix dactylifera*) [12].

Aromatic and medicinal plants are a source of chemical molecules resulting from secondary metabolism, belonging to diverse chemical classes, such as alkaloids, phenols, flavonoids, terpenoids, and steroids [13, 14]. Clove buds (*Jambosa caryophyllus*) are medicinal and aromatic parts of a plant of the Myrtaceae family that contain various constituents, including phenylpropanoids such as carvacrol, thymol, eugenol, and cinnamaldehyde [15].

Essential oils of clove buds (EO-JC) are of particular interest because of their biological activities [15, 16]. EO-JC has broad medicinal properties and applications among other uses, as anticancer, antibacterial, antifungal, analgesic, antiseptic, antidiabetic, antiobesity, anti-inflammatory, antioxidant, antiviral, and aphrodisiac [17–23].

In this study, in order to find a less expensive, effective, and accessible alternative to synthetic insecticides and fungicides, essential oils extracted from clove buds, were characterized by GC-MS, and their insecticidal activity on *C. maculatus*, and antifungal activity, were investigated.

## 2. Materials and Methods

### 2.1. Plant Material

*Jambosa caryophyllus* was collected in Fez City-Morocco in 2021 before being identified by a botanist and given a voucher number JC22/Fez-03. Next, the flower buds of the clove were dried by the use of an oven set to 40°C for 3 days before being ground into a fine powder prior to extraction.

### 2.2. Extraction of EO-JC

Essential oils (EO) of clove buds were obtained by hydrodistillation, using a Clevenger-type extractor [24]. This technique is based on the power of water vapor to transport essential oils. The essential oil yield was determined through the ratio of the mass of flower buds of clove (g) by the mass of essential oil (g) (%).

(1) $$\begin{array}{c} Rd\;\left(\%\right)=\frac{\text{Quantity of oil in g}}{\text{Quantity of plant material in g}}\;*100. \end{array}$$

### 2.3. Composition of EO-JC

Gas chromatography (Ultra GC Trace), linked to a mass spectrometer (Polaris Q), ion trap in electron impact (EI) mode with ionization energy of 70 eV, was used to separate and identify chemical components of EO-JC. This chromatographic apparatus was equipped with a silica capillary column type (CP-SIL5CB) (WCOT fused silica), 60 m in length, 0.32 mm diameter, and 1.25 *μ*m thickness. The column temperature is designed to rise at a rate of 3°C/min from 45 to 290°C. The injector is set to 270°C, while the detector (ionization source) was 200°C. The carrier gas (helium) flow rate was 1 mL/min. The injected sample contains 1.0 *μ*L of diluted oil in hexane. The constituents of EO-JC were identified by matching their mass spectra to those of the library NIST-MS and by calculating the retention index and comparing it with the retention index of literature Adams (2007) [25].

### 2.4. Insecticidal Activity of EO-JC

#### 2.4.1. Conditions of Collection and Rearing of Insects

Chickpea weevils (*C. maculatus*) were maintained on chickpea seeds (*Cicer arietinum*) in transparent boxes of capacity (1 L) covered inside with a transparent fabric to raise the bruchids, deposited in a temperature of about 25 ± 2°C keeping the relative humidity of about 65% ± 5% and a photoperiodic cycle of 14 h (light)/10 h (dark).

#### 2.4.2. Toxicity of EO-JC by the Contact Test

Toxic potency of EO-JC was determined by contact by previously described methods [3, 26, 27], with some modifications. 0.1 kg of chickpeas were infected with five pairs of 0–48 hold insects, placed in plastic containers (0.25 L). For each concentration of EO-JC, we use a perforated top and a thin transparent cloth. After that, using an automated pipette, EO-JC was added to the beans and mixed for 2 minutes. A control group of 0.1 kg chickpeas infected with five pairs of oil-free insects was utilized at the same time. After 48 h of confinement, the mortality of adults was evaluated as described elsewhere [28]. Based on the results obtained in the preliminary tests, a range of concentrations was determined (4, 8, 16, and 32 *μ*L/100 g). The tests were repeated three times for each concentration. Insecticidal potency of EOs was evaluated by daily counting the number of dead *C. maculatus* adults under a stereoscopic microscope (Equation).

(2) $$\begin{array}{c} Pc\;\left(\%\right)=\frac{Po-Pt}{100-Pt}\;*100, \end{array}$$

where *Pc* is the corrected % mortality; *Po* is the observed mortality in the trial, and *Pt* is the mortality in the negative control.

Eggs were counted 12 days after the experiment began, and emerging individuals were counted 30 days later. The decrease in fecundity of females and emerging adults for each concentration of EO-JC was calculated.

(3) $$\begin{array}{c} PR\;\left(\%\right)=\left(1-\frac{Nt}{Nc}\;\right)*100, \end{array}$$

where *PR*  is the reduction rate compared to the control, *Nc*  is the number of eggs or insects in the control, and *Nt* is the number of eggs in the trial.

#### 2.4.3. Toxicity of Essential Oils Tested by Inhalation

Effects of EO-JC on adult *C. maculatus* were determined after exposure via inhalation. Purpose, these adults were placed in 1-liter glass vials, and small cotton masses were suspended with a thread attached to the inside of the lid. Concentrations of 4, 8, 16, or 32 *μ*L EO-JC/L were applied to the cotton with a ProPette™. 10 bruchids (male and female) of *C. maculatus* aged 0–48 hours were placed in each jar with a seal. Three replicates were performed for each dose. In comparison with an untreated control sample, calculation of the mortality rate according to the formula of Abbott is given as follows (Equation) [29]:

(4) $$\begin{array}{c} Pc\;\left(\%\right)=\frac{Po-Pt}{100-Pt}\;*100, \end{array}$$

where *Pc* is the corrected % mortality, *Po* is the mortality in the trial, and *Pt* is the mortality in the negative control.

#### 2.4.4. Repellent Effect of EO-JC

The preferable area technique on the filter paper was used to investigate the repellent effect of EOs against adult *C. maculatus* [29]. Discs were divided into two halves, each having a surface area of 31.80 cm^2^. A volume of 500 *μ*L of each concentration of EO-JC previously prepared in acetone (4, 8, 16, and 32 *μ*L/mL) was evenly distributed for one of the two halves, resulting in doses of 0.016, 0.079, 0.157, and 0.315 *μ*L/cm^2^ per disc, while the other half received only 500 *μ*L of each concentration of EO-JC previously prepared in acetone. The other half received only 500 *μ*L of solvent (negative control). After that, the Petri dishes were covered with Parafilm® for 30 minutes. The number of bruchids on the EO-treated half of the disc was compared to the number of bruchids on the untreated half of the disc. For each experiment, three replicates were placed in the same setting as the insect rearing. Repulsion was measured according to the formula of Lehman [29].

(5) $$\begin{array}{c} Pr\;\left(\%\right)=\frac{Nc-Nt}{Nc+Nt}\;*100. \end{array}$$

*Pr* (%) is the percent repulsion, *Nc* is the number of insects in the negative control area, and Nt is the number of insects in the treatment area with EO-JC.

### 2.5. Antifungal Activity of EO-JC

#### 2.5.1. Evaluation of Fungal Activity

Evaluation of antifungal activity of EO-JC was performed by the use of the disk diffusion method [30]. Petri dishes containing MH (Mueller–Hinton) medium were grown with the three fungal strains and inoculated with *A. niger*, *A. flavus,* and *F. oxysporum* by the agar plot method. Sterile Whatman paper discs (6 mm diameter) were placed in the center of the Petri dish and then impregnated with 20 *μ*L of EO-JC (1 mg/mL) and fluconazole (positive controls) at a dose of 5 mg/mL, whereas the fungal strains were incubated at 37°C. 7 days for *A. niger*, *A. flavus*, and *F. oxysporum*, percent inhibition was evaluated [31, 32]. The percentage of inhibition was calculated according to the mathematical formula:

(6) $$\begin{array}{c} \text{I}\left(\text{\%}\right)=\frac{\text{Dc}-\text{Ds}}{\text{Dc}}*100, \end{array}$$

where I (%) is the percentage of inhibition; Dc is the diameter of the control; Ds is the diameter in the presence of essential oils.

#### 2.5.2. Determination of the Minimum Inhibitory Concentration

The minimum inhibition concentration (MIC) of EO-JC was determined by using the microdilution technique [30]. Microplates were prepared under aseptic conditions, each sterile 96-well microplate was labeled, 100 *μ*L of EO used for the test in 10% (v/v) DMSO was pipetted into the first column of the plate, and 50 *μ*L of sterile ME (malt extract) for the fungal strains was added to all other wells; serial dilutions were made using a multichannel pipette, and finally 30 L after 7 days at 37°C for *A. niger*, *A. flavus*, and *F. oxysporum* [28, 30]. The MIC endpoint is established by direct observation of good development or colorimetric technique 0.2 percent (w/v) [32, 33].

## 3. Statistical Analysis

The results were reported as the mean of three replicates with standard deviations (standard deviation). Shapiro–Wilk tests were used to validate the normality of the variables, and Levene's test was used to examine the homogeneity of variances, and both were performed using GraphPad Prism (version.8.0.1). Analysis of variance (one way-ANOVA) and Tukey's multiple comparison test were used to examine the differences between the means. At a probability level (*p*) 0.05, differences were considered statistically significant.

## 4. Results

### 4.1. EO-JC Extraction Yield

The yield of OE-SA obtained by the four-hourhydro-distillation technique in a Clevenger apparatus was 1.344 ± 0.030%, with a characteristic yellow to clear brown color and aromatic odor.

### 4.2. Chemical Composition of the EO-JC

**Figure 1:**
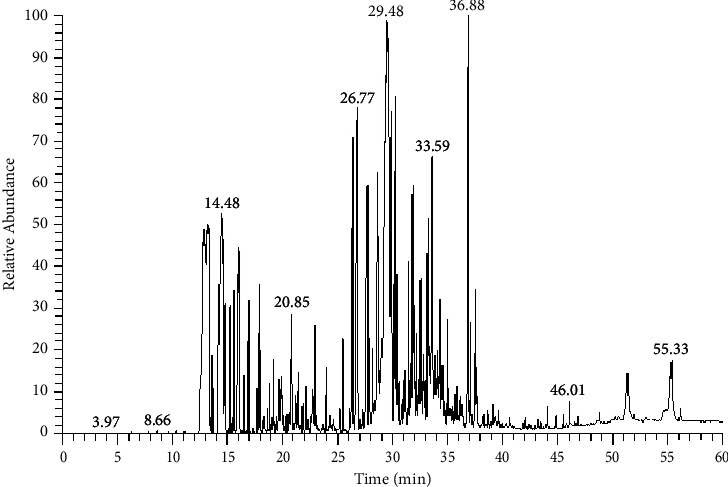
GC-MS chromatogram of essential oils from *J. caryophyllus* clove buds.

Instrumental analysis by GC-MS of the EO-JC showed that the oil consists of 34 compounds representing identified components (Figure 1). These compounds represent more than 99.98% of the total essential oil (Table 1).

### 4.3. Effect of EO-JC on Mortality by Direct Contact with Adult *C. maculatus*

**Figure 2:**
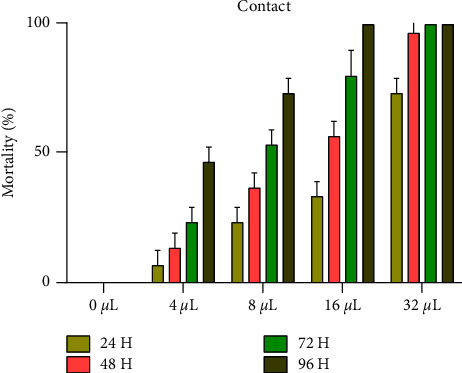
Mortality (%) of adult *C. maculatus* exposed to various concentrations of EO-JC in air.

There was a significant, direct relationship between the concentrations of EO-JC and the mortality of adult *C. maculatus*, and there was also a significant relationship between durations of exposure (Figure 2). During the four observation periods (24, 48, 72, and 96 hours), at the least dose of 4 *μ*L EO-JC/L of air, mortalities at each duration of exposure were 6.68 ± 5.76, 13.33 ± 5.77, 23.33 ± 5.76, and 46.66% ± 5.775, respectively; mortalities at 8 *μ*L EO-JC/L were 20, 36.667 ± 5.7, and 53.33 ± 5.76, 70%, respectively; at exposure to 16 *μ*L EO-JC/L air, mortalities were 33.33% ± 5.777, 56.66% ± 5.76, 80 ± 10, and 100%, respectively. The maximum concentration of 32 *μ*L EO-JC/L air caused complete mortality of approximately 100% during the four durations of exposure.

### 4.4. Effect of Inhalation of EO-JC on Mortality of Adult *C. maculatus*

**Figure 3:**
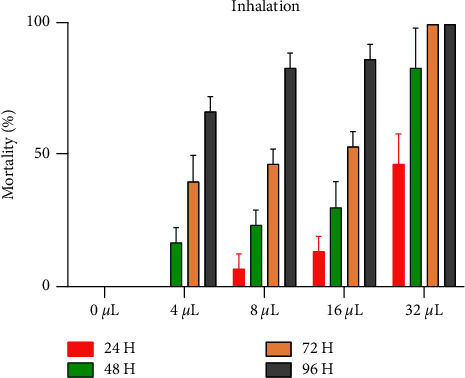
Mortality (%) of adult *C. maculatus* exposed via inhalation to 5 concentrations of EO-JC for 4 durations.

Mortality of adult *C. maculatus* was significantly and directly proportional to the concentration of EO-JC to which they were exposed. Exposure for 24 hours to doses of 0, 4, 8, 16, or 32 *μ*L EO-JC/L of air resulted in mortalities of less than 50%; mortalities were 0.0, 0.0, 13.33 ± 5.77, and 46.66 ± 11.54%, respectively. Exposure for 48 hours to the same concentrations caused mortalities of 0.00, 16.67 ± 5.78, 23.33 ± 5.77, 30 ± 1.0%, and 83.33 ± 15%, respectively. Exposure for 96 hours to 0, 4, 8, 16, and 32 *μ*L EO-JC/L air resulted in mortalities greater 50% with values of 66.66 ± 5.77, 83.33 ± 5.76, 86.67 ± 5.77, and 100%, respectively. Furthermore, the minimum concentration of the essential oil tested to achieve 100% mortality of *C. maculatus* adults was 32 *μ*L/L of air volume after 72 hours of exposure. Relatively, great mortality was observed in adult chickpea bruchids exposed for 96 hours, which showed the potent insecticidal effect of EO-JC. Direct contact to EO-JC for 96 hours resulted in LD_50_ and LD_95_ of 5.51 and 11.05 *μ*L EO-JC/L, air. LD_50_ and LD_95_ values obtained in the inhalation test were greater than those obtained with the contact test, based on statistical analysis. Lethal doses to 50% (LD_50_) and 95% (LD_95_) of individual adult *C. maculatus* were determined for EO-JC (Figure 3). For the inhalation test, the DL_50_ and DL_95_ after 96 hours of exposure were 2.32 and 21.92 *μ*L EO-JC/L, air, respectively.

### 4.5. Effect of EO-JC on Mortality of *C. maculatus* Larvae

**Figure 4:**
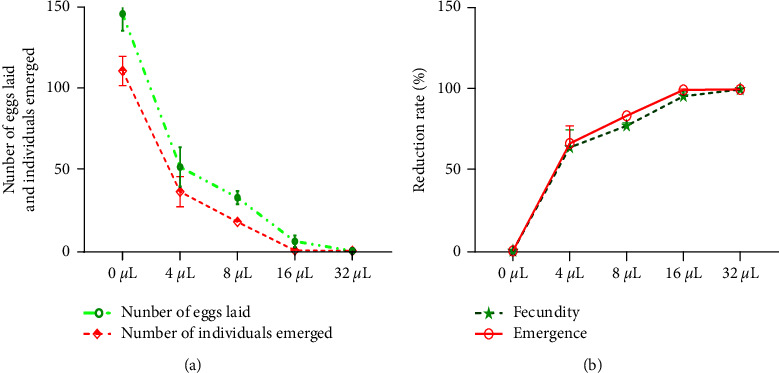
(a) Fecundity and emergence of new individuals after direct contact with 5 doses of EO-JC. (b) Percent reductions of fecundity and emergence after direct contact with five doses of EO-JC.

Exposure of female *C. maculatus* to EO-JC reduced oviposition in a dose-dependent manner (Figure 4(a)), but none of the concentrations of EO-JC completely prevented oviposition. The number of eggs laid per unexposed female *C. maculatus* was 111.0 ± 9.0. At the least concentration of 4 *μ*L EO-JC/100 g), the mean number of eggs per female was 52 ± 12, which represents a decrease of 64 ± 10.8 eggs compared to the negative control (Figure 4(b)). The greatest dose of 32 *μ*L EO-JC/100 g) resulted in a 100% reduction in oviposition compared to the unexposed control females. Exposure to 32 *μ*L EO-JC/100 g also eliminated the emergency of juveniles.

### 4.6. Repellent Activity of the EO-JC

EO-JC was moderately repellent of *C. maculatus* in a dose-dependent manner with *a* maximum repellence of 93.33 ± 11.55% after exposure to 0.503 *μ*L EO-JC/cm^2^ for 60.

### 4.7. Antifungal Activity of EO-JC

EO-JC exhibited antifungal activity against a variety of fungi pathogenic to plants (Table 2). The greatest activity was observed against *A. flavus* (MTCC 9606), with inhibition between 50 ± 2.5 and 87 ± 2.4%. MIC values ranged from 2.9 ± 0.0 to 5.9 ± 0.02 *μ*g EO_JC/mL, which was significantly (*p* ≤ 0.05) greater than that of fluconazole (Table 2).

## 5. Discussion

The main compound in EO-JC is eugenol [17], which has been reported to represent 49.0 to 56% of EO-JC [34, 35]; however, it has been reported to comprise as much as 87.1% of the mass of extracted EOs [33]. However, in the present study, EO-JC constituted only 26.8% (Table 2). Variability in the proportion of EO-JC contributed by eugenol and total yield of EO-JC might be due to various factors such as geographical variations, environmental conditions, physiological variations, genetic factors as well as differences in methods used for extraction of EOs [36, 37]. The insecticidal activity of EO-JC observed against adults of *C. maculatus*, observed in this study, is consistent with the results of previous studies [15].

In the present work, the insecticidal and fungicidal power of EO-JC can be attributed to eugenol and *β*-caryophyllene as they are known for their insecticidal effects, particularly when they act on synapses of herbivorous insects and block the production of neurotransmitters, recorded literature [38–40]. Results of this study indicate that EO-JC can be used for the control of *C. maculatus* populations which is possible. Essential oils of *J. caryophyllus* act by gaseous diffusion [41]. This allows them to reach all interstices in the stored seeds [41]. Thus, they can be used as a fumigant [42, 43]. Like EOs, *J. caryophyllus* powder has been shown to be effective in eliminating individuals of (*C. maculatus*) within 3 days [42, 43]. Furthermore, our results show that treated EO-JC inhibits reproduction of *C. maculatus* F., which is consistent with the results of previous studies [43].

The fact that exposure of adult female *C. maculatus* to EO-JC reduced fecundity might be due to its main component, eugenol, which is a volatile substance contained in the EOs of *J. caryophyllus* [44, 45], and this monoterpene has been evaluated for its toxicity towards *C. maculatus,* which can eventually reach its larvae hidden inside the treated seeds [44–46].

Based on the LD_50_ and LD_95_ of EO-JC against adults of *C. maculatus*, it is likely that it can be used to protect grains from this pest. Insecticidal activity of EOs of *J. caryophyllus* against pests of stored products in particular (*Cicer arietinum*) has been the subject of only a few works such as those of Allali and coauthors [47], but EO-JC has been shown to be effective at controlling several species of insect pests in stored grain [48].

The mechanism of action of these oils is not yet well known. It is therefore important to carry out toxicological studies in order to determine the mode of action of the essential oils and to prevent possible resistance phenomena that might occur during treatments. The fact that the tested EOs were effective on both adults, eggs, and larvae suggests that they act via the respiratory pathway. In *C. maculatus* eggs, the oil vapors act through the egg respiratory tract [49, 50]. This is consistent with reports that the components of essential oils act on the respiratory chain by inhibiting the activity of monooxygenases in the treated insect [51]. It has been reported that the respiratory activity of *C. maculatus* eggs is six times less than that of neoformed larvae. This reflects a low activity of monooxygenases in eggs and therefore a higher tolerance to products that inhibit these enzymes [52]. Eggs, which are classified as a quiescent stage, are more tolerant to synthetic insecticides [53]. Since EO-JC is composed of volatile matter, contact treatment is not applicable. Indeed, contact treatment does not take into account limitations related to evaporation of the active ingredients. Hence, there are difficulties encountered in determining the effective concentration. Fumigation allows large masses of seeds to be treated without the need to mix or move them. Thus, due to its volatility and biodegradation are advantages of using EOs as pesticides [54].

The results of this study that demonstrated broad antifungal activity of EO-JC are consistent with the results of previous studies [55, 56]. It was observed in similar studies that this oil possessed inhibitory activity against *Aspergillus niger* [57, 58]. Similarly, it has been shown that EO-JC has inhibitory effects on strains of *Aspergillus flavus* [59, 60]. The fungicidal activity of *J. caryophyllus* essential oil has also been reported on strains of *Fusarium oxysporum* [56]. The mode of action of the antifungal activity of volatile oils is not well known. However, some compounds in EOs appear to be strong inhibitors of malt amylase and catalase in some fungi [61]. Recently, studies have reported that some volatile oils affect the disruption of the endomembrane system of the fungal cell, including the plasma membrane and mitochondria, as well as inhibition of synthesis of ergosterol, malate dehydrogenase, mitochondrial ATPase, and succinate dehydrogenase [62, 63], all of which result in damage to the cytoplasmic membrane and subsequent leakage of intracellular components such as DNA [64].

## 6. Conclusions

In the present work, essential oils extracted from *Jambosa caryophyllus* clove buds (EO-JC) were investigated for their composition, antifungal, and insecticidal properties. Results showed that EO-JC possessed promising activities, which may be attributed to its richness in eEugenol *β*-caryophyllene and eugenyl acetate. It can be concluded that the essential oils of *Jambosa caryophyllus* clove buds exhibited an interesting insecticidal effect, which is promising as a natural bioinsecticide that can be applied to grain crops to protect the seeds of various pests in crops and stored grains that might be able to be used instead of more expensive and hazardous pesticides.

## Figures and Tables

**Table 1:** Chemical composition of EO extracted from *J. caryophyllus* clove buds.

Peak	RT	Compounds	Molecular formula	Chemical class	RI		Relative Area (%)
					Cal	Lit	
**1**	13.15	*β*-Caryophyllene	C15H25	ST.H	1417	1419	16.03
**2**	15.24	*α*-Thujone	C10H16O	MO.O	1102	1102	0.79
**3**	15.58	3-Carene	C10H16	MO.H	1007	1011	0.76
**4**	16.0	Thymol	C10H14O	MO.O	1285	1290	4.37
**5**	16.49	*α*-Terpinene	C10H18O	MO.O	1015	1017	0.26
**6**	16.93	*α*-Pinene	C10H16	MO.H	936	939	0.8
**7**	17.88	Camphene	C10H16	MO.H	950	954	1.75
**8**	18.84	*β*-Pinene	C10H16	MO.H	974	979	0.24
**9**	19.17	1.8-Cineole	C10H18O	MO.O	1030	1031	0.48
**10**	19.93	Limonene	C10H16	MO.H	1020	1029	1.20
**11**	20.85	Verbenol	C10H16O	MO.O	1139	1141	1.45
**12**	21.43	Terpinene-4-ol	C10H18O	MO.O	1172	1177	0.87
**13**	22.15	Seychellene	C15H24	ST.H	1446	1446	0.37
**14**	22.97	*α*-Copaene	C15H24	ST.H	1377	1376	1.71
**15**	23.95	*α*-Ylangene	C15H24	ST.H	1418	1420	0.33
**16**	25.50	Β-Patchoulene	C15H24	ST.H	1381	1381	0.46
**17**	26.40	Sativene	C15H24	ST.H	1390	1391	3.74
**18**	26.77	Eugenyl acetate	C12H14O3	O	1520	1524	5.83
**19**	27.71	Aristolone	C15H22O	ST.O	1760	1763	3.33
**20**	29.48	Eugenol	C10H12O2	MO.O	1359	1356	26.8
**21**	30.31	Borneol	C10H18O	MO.O	1149	1169	5.0
**22**	31.47	Solanone	C13H22O	O	1360	1361	0.71
**23**	31.85	*β*-Phellandrene	C10H16	MO.H	1029	1029	3.13
**24**	32.54	Myrcene	C10H16	MO.H	991	990	1.04
**25**	33.59	*α*-Humulene	C15H24	ST.H	1452	1454	5.55
**26**	34.33	Cymene	C10H14	MO.H	1425	1426	1.81
**27**	36.88	*β*-Caryophyllene epoxide	C15H24O	ST.O	1665	1667	5.03
**28**	37.12	Bornyl acetate	C12H20O2	O	1285	1288	0.67
**29**	37.51	Terpinyl acetate	C12H20O2	O	1310	1317	1.59
**30**	51.34	Terpinolene	C10H16	MO.H	1080	1088	1.50
**31**	51.44	Eudesmol	C15H26O	ST.O	1660	1663	2.34
**32**	51.55	Trans-verbenol	C10H16O	MO.O	1140	1144	0.01
**33**	54.01	Caranone	C10H16O	MO.O	1200	1200	0.02
**34**	55.33	Linalool	C10H18O	MO.O	1090	1090	0.01

Chemical class							
Monterpene oxygenated (MO.O)							40.06
Monoterpene hydrocarbons (MO.H)							12.23
Other (O)							8.80
Sesquiterpene oxygenated (ST.O)							10.70
Sesquiterpene hydrocarbons (ST.H)							28.19
Total							99.98

RT: retention time; RI: retention index; Cal: calculated; Lit: literature.

**Table 2:** Antifungal activity and minimum inhibition concentrations (MIC) of essential oils extracted from seeds of *J. caryophyllus* (EO-JC) and the synthetic fungicide, fluconazole.

		*Aspergillus niger* (MTCC 282)	*Aspergillus flavus*(MTCC 9606)	*Fusarium oxysporum*(MTCC 9913)
EO-JC	Antifungal activity	50 ± 2.5%^**a**^	89 ± 2.4%^**b**^	56 ± 2.5%^**c**^
	MIC (*μ*g/mL)	2.9 ± 0.02^**a**^	5.9 ± 0.02^**b**^	2.9 ± 0.0^**a**^

Flu	Antifungal activity	8.2 ± 2.0%^**a**^	0.0 ± 0.0%^**b**^	31 ± 0.58%^**c**^
	MIC (*μ*g/mL)	7.1 ± 0.0^**a**^	—	3.1 ± 0.0^**b**^

Means (±SD, *n* = 3) followed by different letters in the same row are significantly different (one-way ANOVA; followed by Tukey's multiple range test, *p* ≤ 0.05). EO-JC: essential oils of *J. caryophyllus* clove buds; Flu: fluconazole.

## Data Availability

Data used to support the findings are included within the article.
